# Genicular Artery embolisation in Patients with Osteoarthritis of the Knee (GENESIS) Using Permanent Microspheres: Long-Term Results

**DOI:** 10.1007/s00270-024-03752-7

**Published:** 2024-05-31

**Authors:** M. W. Little, A. O’Grady, J. Briggs, M. Gibson, A. Speirs, A. Al-Rekabi, P. Yoong, T. Ariyanayagam, N. Davies, E. Tayton, S. Tavares, S. MacGill, C. McLaren, R. Harrison

**Affiliations:** 1 University Department of Radiology, Royal Berkshire NHS Foundation Trust, Reading, UK; 2 Department of Orthopaedics, Royal Berkshire NHS Foundation Trust, Reading, UK; 3 School of Psychology and Clinical Language Sciences, University of Reading, Reading, UK

**Keywords:** Osteoarthritis, Knee, Embolization, Genicular

## Abstract

**Purpose:**

To report the 2-year follow-up of patients with mild-to-moderate knee osteoarthritis (OA) treated with genicular artery embolisation (GAE) as part of the GENESIS study.

**Materials and methods:**

Forty-six patients, median age = 60 (45–83) underwent GAE using permanent microspheres (100–300 μm). Technical success was defined as embolisation of the targeted genicular arteries. Knee Injury and Osteoarthritis Outcome Score (KOOS) and Visual Analogue Scale (VAS) (0–100 mm) were recorded at baseline, 6 weeks, 3 months, 1, 2 years. Contrast-enhanced MRI knee scans were acquired at baseline and 1 year, and evaluated with the Whole-Organ Magnetic Resonance Imaging Score (WORMS). Functional MRI brain imaging and psychometric assessments were undertaken to investigate correlation between neuropsychological phenotypes and clinical outcome. Adverse events were recorded prospectively.

**Results:**

Technical success was achieved in forty patients (87%). Mean VAS improved from 58.63 (SD = 20.57, 95% CI 52.7–65.5) at baselines to 37.7 at 2-years (SD = 26.3, 95% CI 27.0–47.5). Whole and subgroup KOOS were significantly improved at each timepoint with associated reductions in analgesia usage. WORMS analysis demonstrated significant reduction in synovitis (*p* < 0.05) with no cases of osteonecrosis. Self-limiting skin discolouration occurred in four patients. A self-limiting groin haematoma and single case of deep-vein thrombosis due to immobilisation were also recorded. Nine patients subsequently underwent knee arthroplasty with no additional operational complexities identified. Neuropsychometric assessment elucidated a correlation between baseline catastrophising and greater reduction in pain post GAE.

**Conclusion:**

GAE is a safe intervention for mild-moderate knee osteoarthritis, with sustained efficacy at 2 years. These results are promising and justify ongoing controlled trials.

**Graphical Abstract:**

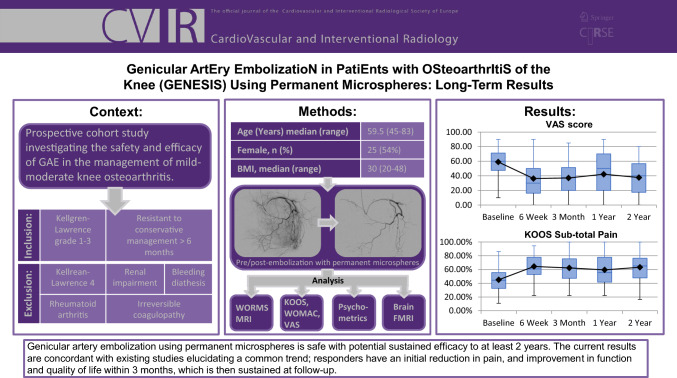

**Supplementary Information:**

The online version contains supplementary material available at 10.1007/s00270-024-03752-7.

## Introduction

Osteoarthritis is an ever-expanding clinical and economic challenge facing healthcare systems around the world [1]. The disease most commonly affects the knee and, even in its less severe forms, can have a substantial impact on patients’ quality of life (QOL) [2]. Patients with mild-moderate disease unresponsive to conservative approaches, who are not suitable surgical candidates, are currently left without further treatment options [3]. Genicular artery embolisation (GAE) is a promising interventional radiology technique that aims to fill this treatment gap [4].

The scientific rationale for GAE is based on low-level inflammation and pro-angiogenic factors promoting the growth of neoangiogenic vessels which infiltrate the knee joint synovium [5]. Due to their shared cellular lineage, neovascular growth is accompanied by neuronal tissue development, which becomes sensitised to nociceptive signals from the surrounding inflammation, and is postulated to be the source of pain in osteoarthritis [6]. GAE exploits the presence of neovessels as an embolisation target, and may reduce regional inflammation, and patient pain.

Initial GAE trials have elicited promising results, however, they have also identified variability in treatment outcomes [4, 7, 8], with no explanation yet proposed for the range of individual differences. One limitation is that, to-date, psychosocial variables have not been considered, and a solely pathophysiological assessment is often ineffective [9]. Specifically, psychometrics that quantifies maladaptive pain cognitions such as the Pain Catastrophising Scale (PCS) [10] are associated with variations in the experience of acute [11] and chronic pain [12]. Pain catastrophising (PC) has been shown to predict outcomes to more invasive surgical interventions such as arthroplasty or mastectomy [13, 14]. PC has been associated with biomarkers associated with the dorsolateral prefrontal cortex and its engagement with pain processing regions of the brain, and may represent an inability to disengage from nociception [15].

This study reports the 2-year follow-up of 40 patients undergoing GAE, of which the interim results of 38 patients were reported previously [16]. Pre-interventional assessment psychosocial data will also be reported, in relation to post-interventional outcomes and underlying neurological mechanisms.

## Materials and Methods

GENESIS is a National Institute for Health Research (NIRH) portfolio single-centre prospective trial which has received full ethical approval (IRAS:237,676, CPMS: 37,741). Patients were recruited from those presenting to the orthopaedic department at Royal Berkshire Hospital, Reading, UK. Inclusion criteria comprised of patients 45 years or older with mild-moderate knee osteoarthritis defined as Kellegren–Lawrence (KL) grade 1–3 on plain X-ray. Patients must have been experiencing knee pain for over 6 months despite conservative management [16]. Potential candidates with rheumatoid or infectious arthritis, renal impairment (eGFR < 45), severe osteoarthritis (KL-grade 4), previous knee arthroplasty, irreversible coagulopathy or a bleeding diathesis were excluded. All patients were reviewed by a consultant orthopaedic surgeon and the interventional radiology research team before being consented and entered into the study.

Forty-six patients attended for GAE as part of the GENESIS study of which, 38 had their interim results reported previously [16]. GAE was performed by two consultant interventional radiologists with 9 and 28 years experience, respectively. The procedural details have been described previously and are included within online supplementation [16]. Embolisation targets were delineated by sites of hyperaemic blush signifying pathological areas of synovium. Prior to embolisation glyceryl trinitrate (GTN) was infused via the microcatheter (in aliquots of 100µg, maintaining a systolic blood pressure of greater than 100mmHg) to promote anterograde flow into the hypervascular tissues. An ice pack was applied to the skin over the embolisation site for 15 min in order to minimise the risk of cutaneous non-target embolisation (NTE) by inducing temporary vasoconstriction [16]. Cone-beam-CT (Philips Allura FD20) was utilised, using 6 ml of 100% iodinated contrast (Iomeron, Bracco, Italy) at 0.3ml/s with a 6 s delay, to confirm the embolisation target and assess the risk of NTE [17]. Once the location and safety of the target was confirmed, 100-300μm Embosphere particles (Merit Medical, USA) diluted in 20mls (300mg/ml) iodinated contrast (Iomeron, Bracco, Italy) were used to embolise the hypervascular synovium in aliquots of 0.1–0.3ml per injection with the aim of “pruning” the neovascular vessels whilst preserving the non-pathological genicular arteries and their innate branches. Post-procedure, patients were observed in the interventional radiology unit for four hours before discharge. Technical success was defined as selective catheterisation and embolisation of the targeted genicular arteries.

The efficacy of GAE was assessed using two well established pain and QOL measures at 6 weeks, 3 months, 1 year and 2 years. Visual analogue scale (VAS) and Knee injury and Osteoarthritis Outcome Scores (KOOS) were collated independently at each timepoint by the interventional radiology research team to avoid the introduction of bias through operator led scoring. KOOS is an extension of the Western Ontario McMasters Universities Osteoarthritis index (WOMAC) allowing comprehensive investigation into the clinical effects of GAE in five domains (pain, other symptoms, function in daily living, function in sport and recreation and knee related quality of life) with 100 indicating no symptoms and 0 severe symptoms. A minimum clinically important difference (MCID) in KOOS scores was defined as 10 [16, 18]. Data were also collected at each timepoint on the occurrence of knee arthroplasty, and knee-specific analgesia usage including paracetamol, NSAIDs and opiate medications. Finally, patient satisfaction questionnaires were designed to ascertain patient-reported outcome measures (PROM) on the GAE procedure. Post-surgical questionnaires were completed by patients prior to discharge on the day of the GAE procedure (Table 1).

**Table 1:** Patient satisfaction questionnaire outcomes on acceptability of GAE

Question	Response scale	Mean response (standard deviation)
1. Did you feel anxious before the procedure?	1(no)-10(very)	2 (3)
2. Did you feel anxious during the procedure?	1(no)-10(very)	3 (3)
3. Did you feel any pain during the procedure?	1(no)-10(very)	4 (3)
4. Was the position of lying on your back uncomfortable?	1(no)-10(very)	3 (3)
5. Was the length of the procedure a problem?	1(no)-10(very)	3 (3)
6. Did you find it difficult to stay still for the procedure?	1(no)-10(very)	2 (2)
7. How did the procedure compare to your expectations?	1(worse)-10(better)	7 (4)
8. How did you find the procedure overall?	1(not unpleasant)-10(very unpleasant)	3 (3)
9. Would you have the procedure again?	1(I wouldn’t mind)-10(I would mind)	3 (3)

Pre- and 12-month post-GAE contrast-enhanced MRI of the knee was utilised to assess synovial hypervascularity alongside potential effects of GAE on joint architecture. MRI sequence details are provided in online supplementation. MRIs were independently assessed by two consultant musculoskeletal radiologists with 9 and 14 years experience who were blinded to patient characteristics and outcome scores. Whole-Organ Magnetic Resonance Imaging Scores (WORMS) were attained to produce standardised imaging assessments [19].

Each patient was offered the opportunity to complete a baseline pre-interventional neuropsychological assessment to investigate potential predictive markers for treatment outcome. Within the pre-interventional assessments, patients completed a baseline psychometric assessment (*n* = 30), and a brain magnetic resonance imaging (MRI) scan (*n* = 17). The psychometric assessment consisted of the Pain Catastrophising Scale (PCS)[50], a 13-item measure scored using a 5-point Likert scale (0 = not at all to 4 = all the time). The other included measures were the Five Factor Mindfulness Questionnaire (FFMQ) [20], a 39-item measure used to quantify an individual’s intrinsic propensity to mindfulness. The State-Trait Anxiety Inventory (STAI) [20] and Becks Depression Inventory (BDI) [22] were used to quantify anxiety and depressive symptoms, respectively. Lastly, sleep quality was quantified using the Pittsburgh Sleep Quality Index (PSQI) [23]. Each of these measures have previously been associated with variations in clinical outcomes following surgical intervention [24–27].

Data for the functional neural MRI component were collected using a Siemens MAGNETOM Prisma 3T scanner (Siemens, Erlangen, Germany), using a 64-channel head and neck coil. The protocol consisted of an initial localiser, followed by a resting-state scan, in which patients were instructed to lie still, and keep their eyes open. Functional data were acquired using a blood-oxygen level-dependent (BOLD) protocol (sequence details provided in online supplementation).

Due to the novelty of the development of a pre-interventional assessment battery for knee embolisation, exploratory analyses were conducted to evaluate the suitability of the psychometric variables for predicting treatment outcomes. Due to the univariate nature of WOMAC, this was selected as the primary dependent variable. Neuroscientific investigations were limited to seed-based whole-brain functional connectivity analyses at rest, wherein the dorsolateral prefrontal cortex was selected as the seed, due to its role in the top-down modulation of pain [15, 28], as well as its specific association with pain catastrophising [29, 30].

### Statistical analysis

KOOS and WOMAC scores were assessed using descriptive statistics and used to create boxplots for each timepoint. Adjusted means, difference from baseline and 95% confidence intervals were calculated with all tests utilising a two-tailed 5% alpha significance level. Inter-observer agreement of WORMS scores was assessed using interclass correlation coefficients (ICC). Ratings were compared using a two-way mixed design, evaluating consistency, with 95% confidence intervals. The results from each WORMS domain were summarised with descriptive statistics. MiniTab v.19.2020.01, Microsoft Excel v. 14.0.7015.1000 and SPSS Statistics v23.0 were used for analyses.

## Results

Forty-six patients (female = 25) with a median age of 59.5 years (range = 45–83) and median BMI of 30 (range = 20–48) underwent GAE with a mean follow-up duration of 17.3 months (range = 3–24 months). The median KL score of the studies population was 3 (range = 1–3), with patients using a variety of methods to manage their pain at enrolment (Table 2).

**Table 2:** Patient baseline characteristics

Age (years) median (range)	59.5 (45–83)
Female, *n* (%)	25 (54%)
BMI, median (range)	30 (20–48)
Analgesia (*n*)	
Paracetamol	20
NSAIDs	20
Opioids	9
*Previous knee-specific treatments*	
Physiotherapy	21
Previous intra-articular Injection	28
Previous arthroscopy	13
Acupuncture	2
PRP injection	1
*Kellgren–Lawrence grade (n)*	
1	5
2	19
3	22

Technical success was 87% as 6 patients were not embolised despite cannulation of the target genicular artery: One due to significant cutaneous supply, three because of anastomotic communication increasing the risk of non-target embolisation and two due to a lack of hyperaemic target [16].

The mean fluoroscopy time was 12.15min (SD7.56). Mean cumulative air kerma was 90.1 (SD 72). Mean volume of embolic injected per patient was 1.97ml (SD1.5). The mean number of genicular arteries embolised per patient was 1.3 (SD = 0.5).

Sixty-seven percentage (31/46) of patients experienced pain on injecting contrast and embolic into the target genicular arteries, which they described as similar to that from their arthritis. Post-embolisation, these patients reported a significant improvement in pain on repeat contrast injection. Of the 40 patients with follow-up data, those with contrast injection pain achieved the MCID more frequently than those who did not (75% and 50% of patients, respectively) and at one-year, contrast injection pain was associated with improved clinical response (*p* = 0.0118); however, it was not significantly correlated with outcome at two-years (*p* = 0.1840).

**Fig. 1:**
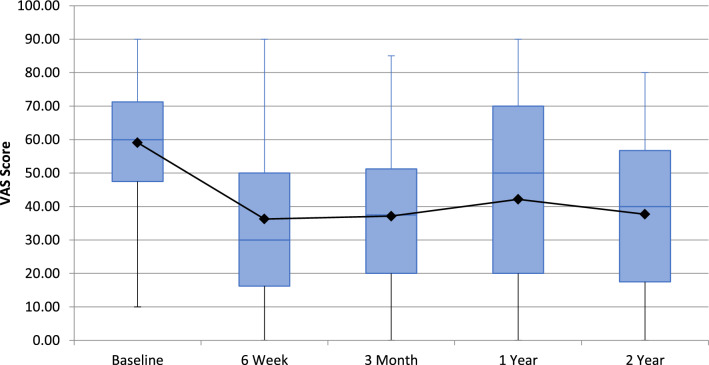
Visual Analogue Scale (VAS) for Pain at follow-up

**Fig. 2:**
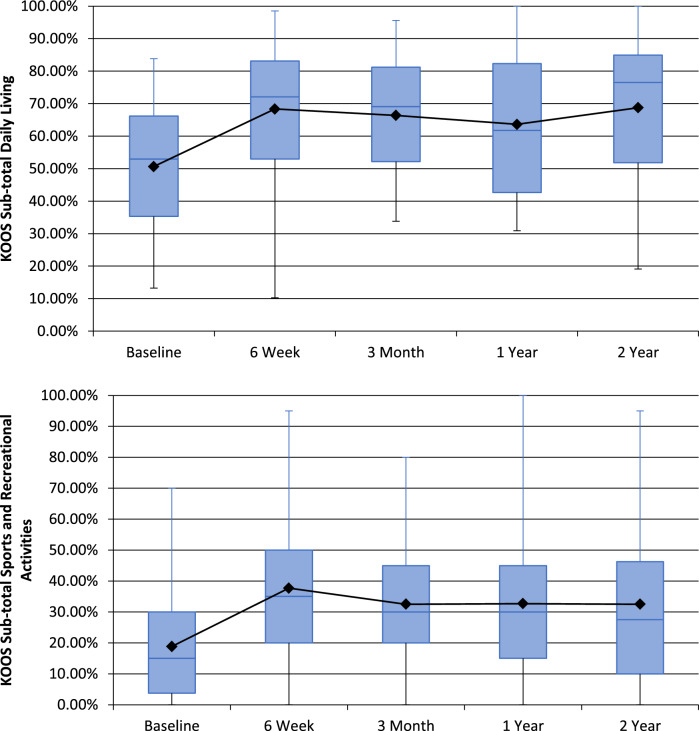

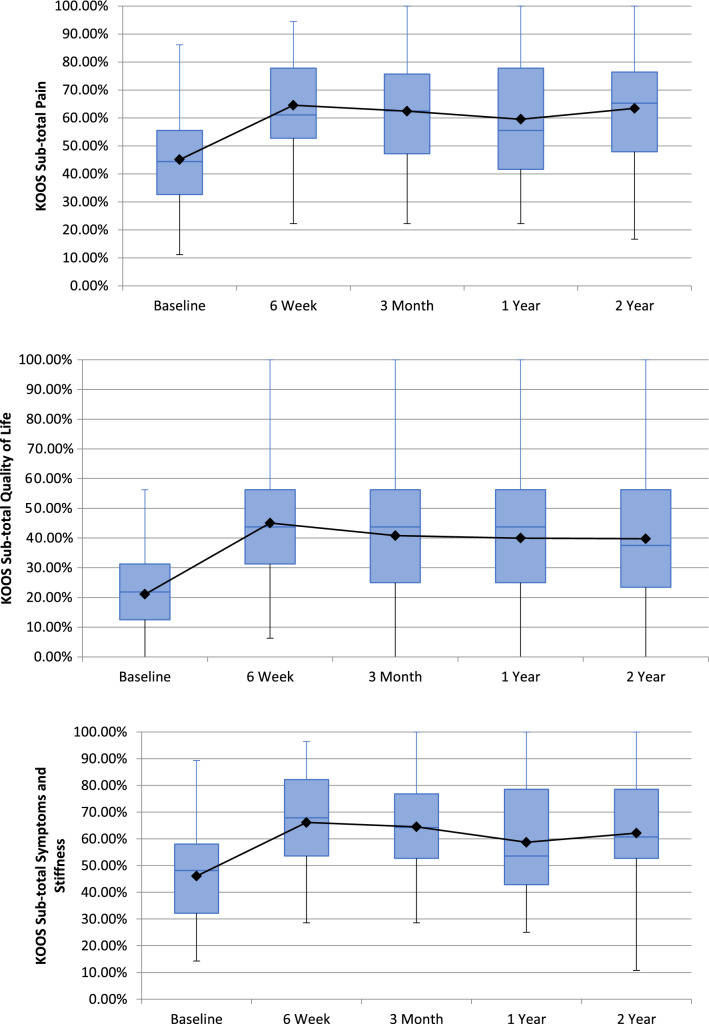
Knee Injury and Osteoarthritis Outcome Score (KOOS) sub-scales

**Fig. 3:**
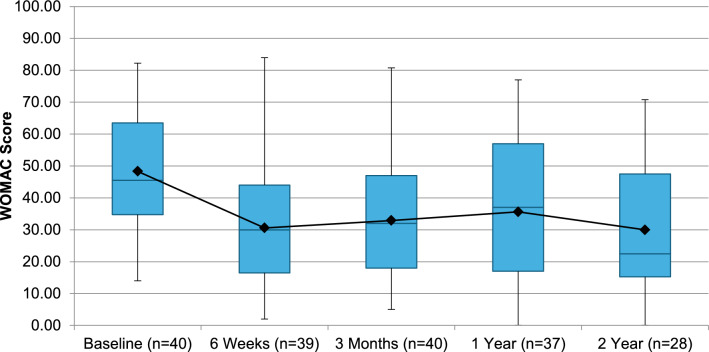
Total western Ontario Mcmaster Universities osteoarthritis index (WOMAC) at follow-up

Of the 40 patients undergoing GAE, 39 reported outcomes at 6-weeks with one patient missing scheduled follow-up. Forty patients completed 3-month follow-up. 37, and 28 patients completed 1 and 2-year follow-up, respectively. Nine patients underwent knee replacement surgery during the study, and 3 were lost to follow-up. There was a significant reduction in VAS from 58.63 (SD = 20.57, 95% CI 52.7–65.5) at baseline to 37.7 at 2-years (SD = 26.3, 95% CI 27.0–47.5) (Fig. 1). Whole KOOS and subscale analysis showed significant improvement from baseline in all variables at 6-weeks (*p* < 0.001), 3-months (*p* < 0.001), 1-year (*p* < 0.05) and 2-years (*p* < 0.05) (Fig. 2). Total WOMAC reduced from a baseline of 48 to 30 at 24-moths (*p* < 0.001) (Fig. 3). At 2-years MCID was achieved in 61%, 39%, 64%, 64% and 57% in KOOS daily living, sports and recreation, symptoms and stiffness, QOL and pain sub scores, respectively.

When evaluating WORMS across the total of each category, consistency between the two reviewers was high (ICC(2,1) = 0.841(0.63–0.94), *p* < 0.001). WORMS scores demonstrated significant progression of cartilage pathology (Mean Change (Δ$\overline{x}$) = 2.8, *p* < 0.05), bone attrition ((Δ$\overline{x}$ = 0.3, *p* < 0.05) and osteophyte development ((Δ$\overline{x}$ = 3.6, *p* < 0.05). As with the interim analysis, synovitis improved post embolisation at 1-year MRI follow-up ((Δ$\overline{x}$ = − 0.5, *p* < 0.05) (Table 3). There was no significant change in meniscal or ligamentous disease, bone cysts or marrow oedema severity. There were no cases of osteonecrosis.

**Table 3:** Whole-Organ Magnetic Resonance Imaging Score (WORMS) of the knee in osteoarthritis

WORMS analysis	Baseline (mean)	Baseline SD	1-year mean	1-year SD	*p*-value
Cartilage	18.5	10.5	20.9	10.6	0.003
Marrow	4.7	3.6	5.4	3.7	0.06
Bone cysts	1.1	1.3	1.4	1.8	0.17
Bone attrition	0.9	1.5	1.2	1.7	0.02
Osteophytes	17.4	14.4	21.1	15.5	0.001
Menisci	2.8	2.3	2.9	2.4	0.61
Ligaments	0.09	0.3	0.09	0.3	1
Synovitis	1.5	0.9	1.0	0.6	0.003
Total	47.0	25.8	54.0	28.1	0.0001

Using the methodology employed in the interim analysis, a pooled analysis of the patient satisfaction questionnaire resulted in 77% of responses returning a positive response (*p* < 0.05) [16].

At enrolment into the study, several patients were using regular analgesia: Paracetamol (*N* = 20), NSAIDs (*N* = 20) and opiates (*N* = 9) for the management of their knee pain. At the 2-year timepoint paracetamol ((Δ$\overline{x}$ = − 9, *p* = 0.056) and NSAID ((Δ$\overline{x}$ = − 11, *p* = 0.05) analgesia had a borderline significant decrease in usage. There was no significant decrease in the usage of opiate analgesia ((Δ$\overline{x}$ = − 1, *p* = 0.77). Nine patients underwent knee arthroplasty despite technically successful GAE to manage their ongoing symptoms. The average time from GAE to surgery was 18months. Median KL-grade in those patients having knee arthroplasty was 3. Mean BMI = 30. There were no technical issues at surgery when undergoing knee arthroplasty following GAE. Despite technically successful knee surgery, 2 of the 9 patients have ongoing severe knee pain and functional limitation.

All adverse events were recorded prospectively in line with the Cardiovascular and Interventional Radiological Society of Europe (CIRSE) Quality Assurance Document and Standards for Classification of Complications [31]. Self-limiting skin discolouration was the most encountered adverse event, occurring in four patients (10%) with all cases recovering within 3 weeks (grade-3). One patient experienced a small, self-limiting haematoma of the groin (grade-2). One patient undergoing GAE developed a popliteal deep vein thrombosis (DVT) 15-days post-intervention (grade-3).

At baseline, pain catastrophising was significantly correlated with all psychometric baseline measures. PCS was significantly associated with poorer sleep quality (PSQI; *r*(29) = 0.40, *p* = 0.03), lower trait mindfulness (FFMQ; *r*(28) = − 0.45, *p* = 0.02) and higher depression (BDI; *r*(29) = 0.41, *p* = 0.03) and anxiety (STAI; *r*(29) = 0.53, *p* = 0.003). Higher catastrophising scores were also associated with higher baseline pain ratings via the WOMAC (*r*(30) = 0.39, *p* = 0.03) and KOOS_pain_ subscale (*r*(30) = − 0.45, *p* = 0.01).

**Fig. 4:**
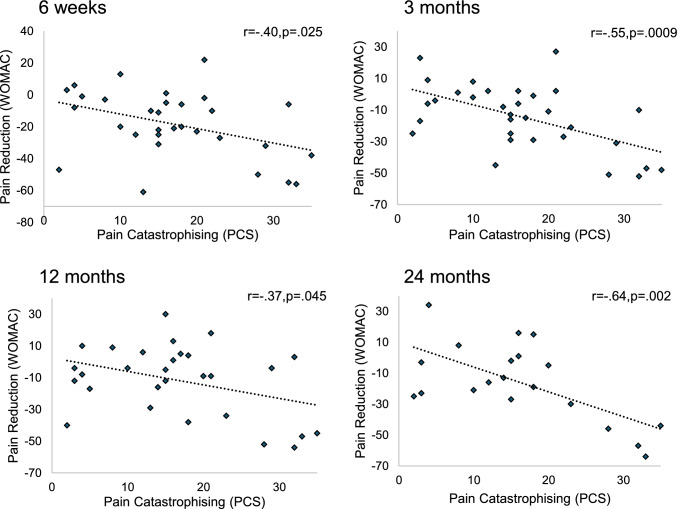
Pain Catastrophising with Total WOMAC

Interestingly, results indicated that those who demonstrated higher levels of catastrophising at baseline experienced the greater reductions in pain post-embolisation (Fig. 4). Pain catastrophising significantly predicted reductions in pain, as reported via WOMAC, at 6-weeks.

**Fig. 5:**
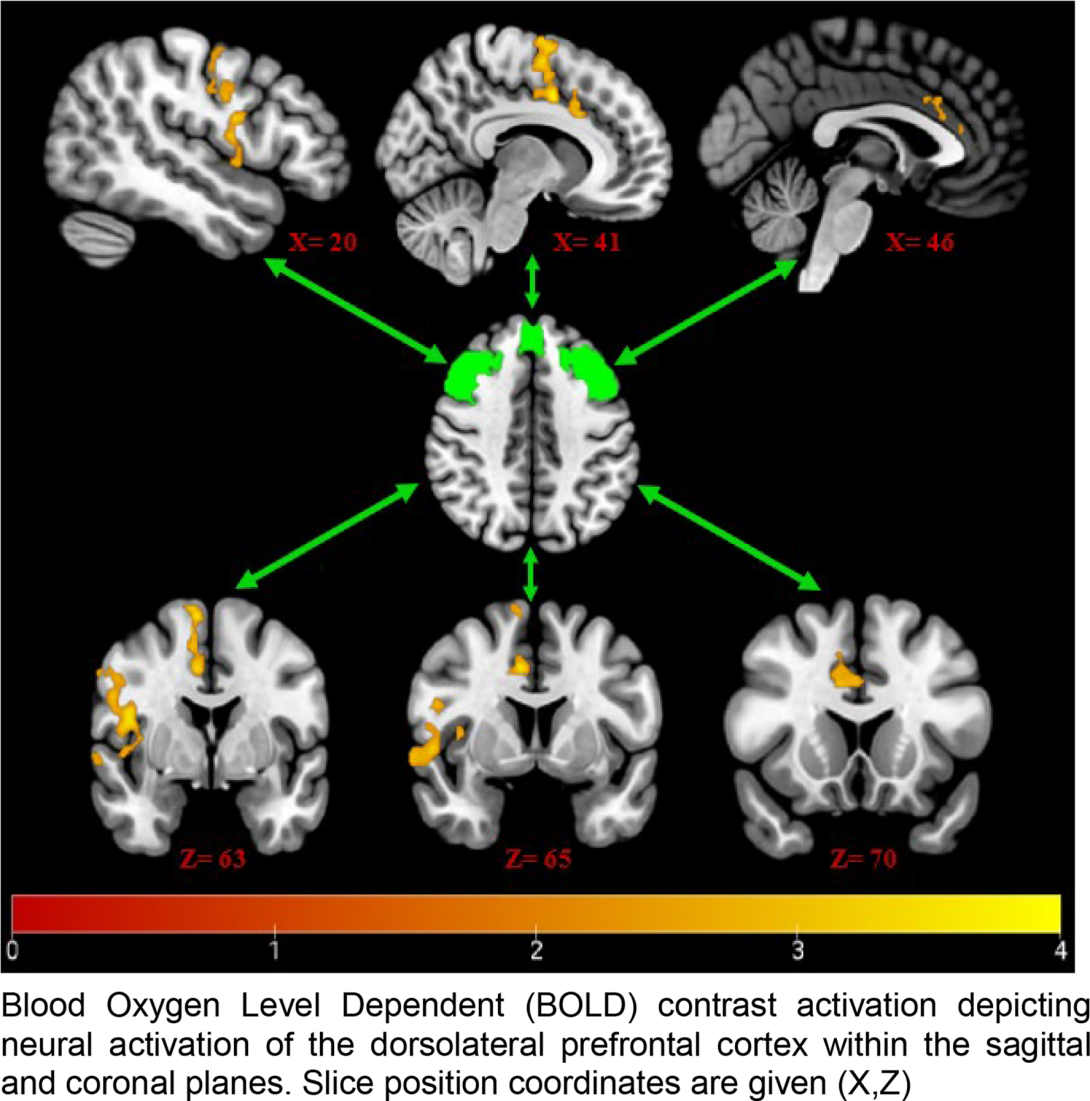
fMRI data of Neuroconnectivity

Functional connectivity analysis of resting-state data revealed higher PCS scores were associated with higher connectivity between the dorsolateral prefrontal cortices and two clusters extending across regions associated with the processing of pain, namely the somatosensory, motor, premotor and anterior cingulate cortices (Fig. 5).

## Discussion

There is increasing literature supporting the use of GAE for the treatment of patients with mild-to-moderate knee OA. These findings coalesce with previous literature highlighting that GAE can be performed safely to produce meaningful reductions in pain and functional limitation [16, 32–42]. This study expands on previous findings by presenting prospective data on patients treated with permanent microspheres with clinical and imaging follow-up to 24 months. The duration of follow-up is crucial for two reasons. Firstly, it provides support to the intervention’s robustness without the need for retreatment to maintain symptomatic control. Secondly, it provides evidence that GAE can achieve the MCID well beyond the expected duration of the placebo effect, providing support for ongoing work to produce control data to further elucidate treatment effect [43].

The safety profile of the results is consistent with previous studies, with non-target cutaneous embolisation being the most common adverse event. There were no cases of skin ulceration, and all skin changes resolved spontaneously within 3 weeks. Our rate of cutaneous non-target skin embolisation is lower than the comparable studies using permanent particles (*n* = 4, 10%) [32, 33, 38, 39]. Using 100-300micron permanent particles, application of an icepack and confirming position with cone-beam CT may all minimise this common complication. We report one serious complication of a DVT following GAE, which has not previously been reported. The venous thrombus was not directly related to GAE. Moreover, it was related to immobility in the post-operative period. This complication highlights the importance of early mobilisation post GAE.

PROMS analysis demonstrates that GAE is highly acceptable to patients, with individuals experiencing low levels of pre- and intra-procedural anxiety alongside manageable levels of pain and discomfort (Table 1). These data also indicate that patients would be open to having repeated procedures, which is critical, as contralateral treatment and ipsilateral re-embolisation will likely become more commonplace with the procedures continued development.

Pain on injection has been shown to correlate with improved outcomes from GAE at one year, but the relationship did not hold to 2 years. The initial hypothesis for pain prior to injection at a pathological level was the role that vascular changes and nerve sensitisation play within the synovium in osteoarthritis. It was hoped that it may provide prognostication when advising patients on the likelihood of clinically significant pain improvement post procedure; however, the lack of sustained correlation questions the mechanism and utility of this finding.

Of the 40 patients undergoing GAE, nine subsequently underwent total knee replacement. Crucially, these patients were followed up post-surgery to identify any intraprocedural issues experienced by the operating surgeons. No adverse events or additional operational complexities were identified in the post GAE cohort. This provides further support for the procedure’s utility in those with mild-moderate disease as it appears that embolisation can be utilised in this cohort without being prohibitive of further surgical intervention in cases of disease progression.

WORMS analysis confirmed the interim analysis, revealing consistent significant improvement in synovitis post GAE. This is an important finding as it supports the scientific rationale that neoangiogenesis can be targeted to reduce inflammation. Furthermore, synovitis has been correlated with severity of pain, cartilage loss and a future risk of TKA in previous studies [44, 45]. MRI evaluation at 1-year post GAE demonstrated worsening cartilage pathology, bone attrition and increased osteophytes. Although there has been significant worsening of these scores post GAE, progression of osteoarthritis is expected over a 12-month period [46]. It is also important to note that the severity of cartilage pathology on MRI has been shown by several trials not to be correlated with experienced pain [47]. The longitudinal progression of osteoarthritis on MRI following GAE should be further clarified in controlled studies [43].

This study demonstrated decreased usage of paracetamol and NSAIDs post GAE, signifying the pain controlling effects of the procedure. Interestingly, there was no change of usage in the opioid taking population. This may represent a distinct population of patients with chronic pain that are challenging to treat and may represent a poor prognostic factor for clinical success following GAE.

The learning curve experienced in this trial reveals the complexities underlying safe and successful performance of GAE and is reflected in this studies technical success rate of 87%. The most important factor in this is a thorough knowledge of genicular vascular anatomy, including the myriad of anastomoses and non-target tissues supplied by these vessels. The most significant anastomoses to appreciate are DGA-SMGA, IMGA-medial sural and genicular artery-MGA as these pose the risk of NTE to the foot, tibial nerve and deep knee structures, respectively [17]. Initially, the lack of description of these anastomoses led to procedural abandonment to ensure patient safety. Now, improved anatomical understanding and the application of intraprocedural cross-sectional imaging allows GAE to be safely undertaken in more morphologically complex cases. Alongside this, embolisation endpoint is another important area of developing understanding with early signals that complete embolisation of a diseased territory is important for clinical success [35].

Neuropsychological experiements identified the predictive capability of PCS as a pre-interventional psychosocial marker. As PCS has previously been associated with poor outcomes following invasive surgical interventions [13], our data indicating higher catastrophising was associated with better surgical outcomes was unexpected. However, the majority of prior studies investigated surgical interventions with longer periods of postsurgical pain, for more severe pathological diagnoses and incur more explicit tissue damage (such as total knee arthroplasty, mastectomy or spinal surgery) than GAE for mild-to-moderate osteoarthritis [14]. These studies identify that catastrophising within the first few weeks is likely to be severely impacted by postoperative pain. GAE is associated with a short postoperative recovery time, and is minimally invasive in comparison to (i.e.) arthroplasty for severe osteoarthritis.

Our findings may suggest that, for embolisation, the predictive influence of catastrophising could represent patients who experience a bi-fold improvement. Firstly, a reduction in their pain, which may in turn reduce the detrimental influence of catastrophising. The neurological data suggest that those who catastrophise at baseline are associated with higher functional connectivity between pain modulatory and processing regions of the brain, perhaps representative of frequent dependency on pain modulation [15]. These patients could be benefitting from a successful reduction in nociceptive signalling via embolisation and an overreliance on pain modulatory circuitry. It is unclear whether this would also be associated with a decrease in catastrophising alongside, as neuropsychological data was only collected at baseline, and needs future investigation to clarify.

The limitations of the current analysis are the relatively small sample size, and lack of experimental control group. As with all treatments designed to treat pain, the placebo effect requires consideration. Two previous randomised controlled trials have reported improved clinical success in patients undergoing complete GAE of diseased territories when compared to a sham procedure [33, 35]. The GENESIS 2 trial is a randomised sham-controlled trial that is currently recruiting and builds on the experience gained from the current work [43]. GENESIS 2 aims to formally consider the placebo effect inherent with GAE, which was not appraised in the current pilot study.

## Conclusion

Genicular artery embolisation using permanent microspheres is safe with potential sustained efficacy to at least 2 years. The current results are concordant with existing studies elucidating a common trend; responders have an initial reduction in pain, and improvement in function and quality of life within 3 months, which is then sustained at follow-up. GAE was particularly beneficial for patients who experience high pain catastrophising at baseline. This phenotype has previously been identified as vulnerable to poor outcomes following more invasive surgical interventions, and GAE may represent a valuable alternative in managing these mild-moderate OA patients. Future studies should investigate optimal embolic materials, longitudinal imaging changes, biomarkers and neuropsychological phenotypes in order to elucidate the ideal patient population for GAE. More work is also required on the severity of OA and outcome following GAE, as related to the pathogenesis of OA.

## Supplementary Information

Below is the link to the electronic supplementary material.

Supplementary file1 (DOCX 14 KB)
