# Correction to: Genicular Artery Embolisation in Patients with Osteoarthritis of the Knee (GENESIS) Using Permanent Microspheres: Long-Term Results

**DOI:** 10.1007/s00270-024-03868-w

**Published:** 2024-09-19

**Authors:** M. W. Little, A. O’Grady, J. Briggs, M. Gibson, A. Speirs, A. Al-Rekabi, P. Yoong, T. Ariyanayagam, N. Davies, E. Tayton, S. Tavares, S. MacGill, C. McLaren, R. Harrison

**Affiliations:** 1 University Department of Radiology, Royal Berkshire NHS Foundation Trust, Reading, UK; 2 Department of Orthopaedics, Royal Berkshire NHS Foundation Trust, Reading, UK; 3 School of Psychology and Clinical Language Sciences, University of Reading, Reading, UK

**Correction to: Cardiovasc Intervent Radiol** 10.1007/s00270-024-03752-7

The original article states in “Results” section: “At 2-years MCID was achieved in 100%, 71%, 100%, 82% and 100% in KOOS daily living, sports and recreation, symptoms and stiffness, QOL and pain sub scores, respectively.”

This sentence has been corrected to: “At 2-years MCID was achieved in 61%, 39%, 64%, 64% and 57% in KOOS daily living, sports and recreation, symptoms and stiffness, QOL and pain sub scores, respectively.”

The original article has been corrected.

