# Supplementary material for: Genicular Artery embolisation in Patients with Osteoarthritis of the Knee (GENESIS) Using Permanent Microspheres: Long-Term Results
Source: Cardiovasc Intervent Radiol. 2024 May 31;47(12):1750–62. doi: 10.1007/s00270-024-03752-7 (PMC11621196; doi:10.1007/s00270-024-03752-7)
Supplement: Supplementary file 1 — Supplementary file1 (DOCX 14 KB) [file 270_2024_3752_MOESM1_ESM.docx]

**Genicular Artery Embolisation Procedural Details**

Anterograde access of the common femoral artery was secured via the insertion of a 4-french vascular sheath (Cordis Medical, USA) under ultrasound guidance. Access to the distal superficial femoral artery was achieved using a hydrophilic guidewire (Terumo, Japan) and 4F glide Cobra Performa catheter (Merit Medical, USA). From this position digital subtraction angiography (DSA) was performed using 300mg/ml iodinated contrast (Iomeron, Bracco, Italy). Genicular arterial catheterisation targets were chosen based on patients’ pain distribution on prior clinical examination, and areas of synovitis on pre-procedural contrast-enhanced MRI. Genicular arteries were selected using a Fathom 14 guidewire (Boston Scientific, USA), following which a straight-tip microcatheter (2.0-2.9- French Pursue, Merit Medical, USA) was introduced through the parent catheter into the designated genicular artery.

**MRI knee Protocols**

Proton density fat-saturation (PDFS), sagittal (Slice-thickness (ST) 2.5, TR 4177 ms, TE 30 ms, field of view (FOV) 144, matrix 360 × 286), axial (ST 3, TR 4589, TE 30, FOV 144, matrix 360 × 275), and coronal sequences (ST 2.5, TR 4848, TE 30, FOV 160, matrix 516 × 366) were acquired using a Philips Ingenia 3T MRI scanner. Contrast-enhanced axial T1-FS (ST 3, TR 578, TE 20, FOV 144, matrix 344 × 276), and sagittal T1-FS (ST 2.5, TR 697, TE 20, FOV 144, matrix 344 × 276) sequences were also acquired.

**Neuro-psychology MRI Brain Protocols**

Functional data were acquired using a blood-oxygen level-dependent (BOLD) protocol with a T2*-weighted gradient echo planar imaging sequence (TR= 1000ms, TE= 30s, slice thickness= 2mm, FA= 90°, 256x256 matrix, voxel size= 2x2x2, FOV=256mm). To reduce the impact of field inhomogeneity, an initial 5 volumes were discarded, and subsequently 600 volumes were acquired, equally a total scan time of 10 minutes and 28 seconds. Following the resting-state, two field maps were collected, followed by a 5-minute T1-weighted inversion recovery fast gradient echo high-resolution anatomical scan (TR= 2300ms, TE= 2.29ms, slice thickness= 0.94mm, FA=8°, 256x256 matrix, voxel size= 0.9x0.9x0.9, FOV=240mm).
